# Differential Regulation of Proinflammatory Mediators following LPS- and ATP-Induced Activation of Monocytes from Patients with Antiphospholipid Syndrome

**DOI:** 10.1155/2015/292851

**Published:** 2015-02-15

**Authors:** Anush Martirosyan, Martin Petrek, Zdenka Navratilova, Armen Blbulyan, Anna Boyajyan, Gayane Manukyan

**Affiliations:** ^1^Department of Pathological Physiology, Faculty of Medicine and Dentistry, Palacky University, 775 20 Olomouc, Czech Republic; ^2^Group of Molecular and Cellular Immunology, Institute of Molecular Biology, National Academy of Sciences, 0014 Yerevan, Armenia; ^3^Department of Obstetrics, Institute of Perinatology, Obstetrics and Gynecology, 0078 Yerevan, Armenia; ^4^Laboratory of Macromolecular Complexes, Institute of Molecular Biology, National Academy of Sciences, 0014 Yerevan, Armenia

## Abstract

Antiphospholipid syndrome (APS) is an acquired autoimmune disorder characterized by recurrent thrombosis and pregnancy morbidity in association with the presence of antiphospholipid antibodies. Growing evidence supports the involvement of monocytes in APS pathogenesis. Inflammatory activation of monocytes promotes thrombus formation and other APS complications. However, mechanisms underlying their activation are poorly investigated. We aimed to determine transcriptional activity of monocytes after exposing them to low concentrations of lipopolysaccharide (LPS) and LPS + adenosine triphosphate (ATP) using comparative qRT-PCR. The results showed that LPS significantly increased transcriptional levels of TLR2, IL-23, CCL2, CXCL10, IL-1*β*, and IL-6 in APS cells, while, in cells from healthy donors, LPS resulted in IL-6 and STAT3 elevated mRNAs. Double stimulation of the cells resulted in decreased mRNA levels of NLRP3 in monocytes isolated from healthy donors and CCL2, IL-1*β* in APS cells. By contrast, TLR2 mRNAs were elevated in both investigated groups after culture of the cells with LPS + ATP. Thus, the findings indicate increased sensitivity of APS cells to LPS that may contribute to thrombus formation and enhance development or progression of autoimmune processes. Low concentrations of ATP diminish LPS-induced inflammatory state of APS monocytes which might be a potential mechanism which regulates inflammatory state of the cells.

## 1. Introduction

Antiphospholipid syndrome (APS) is a systemic autoimmune disorder which is characterized by the persistent presence of pathogenic antiphospholipid antibodies (aPL) directed against cellular phospholipids and phospholipid-binding proteins [1]. The most characteristic features of the syndrome are recurrent thrombosis and pregnancy complications, including miscarriages [2]. The origin and role of the heterogeneous family of aPL in APS have long been appreciated, and the mechanisms of their pathogenicity remain a subject of intense research. Several mechanisms have been proposed for how autoantibodies cause the disease, including interaction with cell surface receptors, such as TLR/IL-1 receptor family members [3], annexin A2 [4], and activation of the complement complex in vessels [5]. The proposed mechanisms involve direct activation of immune cells, particularly monocytes, by interaction with membrane phospholipids [6]. It has been demonstrated that aPL might induce thrombus formation through* in vitro* activation of endothelial cells [7], platelets [8], and monocytes [8, 9]. Growing evidence supports the importance of monocyte activation in APS pathogenesis. Transmigration of activated monocytes through the venous and arterial walls is a key event in APS thrombus formation [10]. It is well known that important mechanism of hypercoagulability in APS is increased expression of tissue factor (TF) [11], as well as increased production of proinflammatory cytokines, chemokines, and adhesion molecules by activated monocytes which leads to the attraction of additional monocytes and T helper cells to the sites of inflammation [12, 13]. Further accumulations of immune cells at the sites of extravascular inflammation focus and amplify the inflammatory response.

It has become apparent that monocytes play a central role not only in innate responses but also in priming and maintenance of adaptive responses [14]. Dysregulation in monocyte function may affect cellular processes in the adaptive system and vice versa. Monocytes are crucial players in defense against infection and exert a wide range of functions including regulation of the immune response, wound healing and repair, scavenging of senescent cells, and remodeling of tissues [15]. The activation of monocytes is elicited by the recognition of self- and non-self-stimuli mediated through pattern recognition receptors. Toll-like receptors (TLRs) are membrane receptors responsible for the self- and non-self-recognition of evolutionarily conserved structures on pathogens, termed pathogen-associated molecular patterns (PAMPs) such as lipopolysaccharide (LPS) [16, 17]. TLR activation leads to expression of a range of inflammatory genes, including IL-1*β* [18]. IL-1*β* lacks a secretory signal peptide and is secreted through a nonclassical pathway [19], and there is a need for a secondary stimulus, such as endogenous adenosine triphosphate (ATP), to promote posttranslational processing of IL-1*β* [20, 21]. This process requires the assembly of the cytoplasmic multiprotein complex called an inflammasome [22], responsible for the caspase-1-dependent cleavage and release of the biologically active, mature IL-1*β*. The most studied one is the NLRP3 (NOD-like receptor family, pyrin domain containing 3) inflammasome which may be triggered by diverse stimuli, including PAMPs, toxins, or damage-associated molecular patterns (DAMPs) [23].

Although inflammation has always been considered as an important mechanism in APS, only few data are available on the molecular processes that lead to the inflammation. It was shown that excessive sensitivity of inflammasome components to external stimuli in genetically predisposed individuals may cause abnormal activation of immune cells, particularly monocytes [24]. Besides well-studied mechanisms of monocyte activation by aPL, other mechanisms are likely to contribute to the process of abnormal monocyte activation. Altered sensing of the innate immune cells to bacterial products and/or “danger” signals may contribute to the ongoing inflammation in APS [25]. Once triggered, monocytes can cause dramatic changes to tissues and organs in the host. Therefore, the aim of the present study was to evaluate the effects of LPS and LPS + ATP on proinflammatory signaling in monocytes from young nonpregnant women with a history of recurrent pregnancy loss and healthy subjects* in vitro*. Thus, we addressed the questions whether there is an altered sensitivity of exogenous bacterial signal (LPS) by monocytes from patients with APS and to analyze the contribution of an endogenous damage signal (ATP) to this process.

## 2. Materials and Methods

### 2.1. Patients

Eleven women suffering from recurrent miscarriages and antiphospholipid syndrome (mean age 30 ± 5.6 years) were selected for this study. APS patients were classified at the Institute of Perinatology, Obstetrics and Gynecology according to the following criteria: (i) women with a history of two unexplained consecutive spontaneous pregnancy losses in the first and second trimesters and (ii) anti-cardiolipin antibodies (IgG ≥ 40 GPL units) and/or anti-*β*2 glycoprotein-I (IgG ≥ 40 GPL units) and/or positive lupus anticoagulant antibodies present in plasma at two separate time points. None of the women met the criteria for systemic lupus erythematosus or any concomitant systemic autoimmune disease. All studied women had pregnancy loss at least a month before blood collection. Nine healthy women (mean age of 29 ± 8.5 years) without a positive family history of APS, autoimmune diseases, and thrombosis were also invited to participate in the study as a control group. All participants in this study had provided written informed consent. The study was approved by the Ethics Committee of the Institute of Molecular Biology of the NAS RA (IRB IORG0003427).

### 2.2. Preparation of Peripheral/Circulating Monocytes

Blood samples were collected from each subject into tubes treated with EDTA. Monocyte isolation by the adherence method was performed as described previously [26]. Briefly, peripheral blood mononuclear cells (PBMCs) were isolated immediately after collection using Ficoll density gradient centrifugation (Life Science, Sweden). After isolation, PBMCs were cultured in 2 mL of RPMI 1640 medium supplemented with 10% fetal bovine serum, and allowed to adhere to the surface of the plastic tissue culture flasks in an incubator at 37°C for 2 hours. Nonadherent cells were removed and the adherent cells were washed twice with PBS. This method yielded >75% pure monocytes as assessed by flow cytometry using anti-CD14 IgG (BioLegend, UK).

Monocytes (1 × 10^6^ cells/mL) were cultured separately at 37°C in RPMI 1640 supplemented with 10% fetal bovine serum, 2 mmol/L glutamine, 5 mM HEPES in the absence or presence of 10 ng/mL LPS (*Escherichia coli* 026:B6; Sigma), or 10 ng/mL LPS + 100 *μ*M ATP*γ*-S (adenosine 5′-[*γ*-thio]triphosphate) (Sigma) in a total volume of 1 mL for 4 h. After the culture period or directly after isolation, cells were washed once with cold PBS and stored in 150 *μ*L RNAlater (Qiagen, Germany) at −20°C until use.

### 2.3. RNA Extraction and RT-qPCR

Total RNA was isolated from all samples using High Pure miRNA Isolation Kit (Roche) according to the manufacturer's instructions. All samples were treated with RNaseOUT Recombinant Ribonuclease Inhibitor (Invitrogen, USA) and kept at −80°C. cDNA synthesis was performed using Transcriptor First Stand cDNA Synthesis Kit (Roche Applied Science, USA); conditions of reverse transcription and primer concentrations were set according to the manufacturer's recommendations.

PCR mixes were prepared as follows: 5 *μ*L of cDNA calculated on input total RNA for each individual gene was added to 20 *μ*L of PCRMix (ABgene, UK). The final concentrations of each component are as follows: 900 nM of each of the sense and antisense primers; 3.5 mM of MgCl_2_; 200 *μ*M of each of the dNTPs, 1U Thermo-Start TAQ polymerase, and 1x Thermo-Start Buffer (ABgene). cDNA was stored at −20°C before further use. After initial denaturation (one cycle at 94°C for 15 min), 40 cycles of amplification (94°C for 45 s, 60°C for 30 s) were performed on the Rotor-Gene 3000 system (Corbett Research, Australia). RT-negative reactions were performed for standards and sample RNA.

The primer sequences, probes, and amplicon sizes for investigated genes are listed in Table S1 (see Supplementary Material available online at http://dx.doi.org/10.1155/2015/292851). The expression of the housekeeping gene RPL32 was used for normalization of target gene expression in monocytes. A human universal reference RNA (Stratagene, USA) was used as a calibrator. The calibrator with total concentrations of 10, 5, and 2.5 ng of RNA/reaction was assessed once and 1.25 ng of RNA/reaction was assessed in triplicate for each run. Relative expression was calculated using the second derivative method (Rotor-Gene software 6.1.71, Corbett Research).

### 2.4. ELISA

Enzyme-linked immunosorbent assay (ELISA) kits were used to measure the levels of IL-1*β* and IL-10 (BioLegend, UK) in the culture supernatants. The assays were performed following the manufacturer's specifications, and absolute cytokine levels were calculated based on comparison to assay performance in the presence of known quantities of recombinant cytokine standards. The detection limits of ELISA were 2.0 and 3.9 pg/mL for IL-1*β* and IL-10, respectively. Results obtained with supernatants were expressed as pg/mL.

### 2.5. Statistical Analysis

Statistical analyses were performed using the statistical software Graph Pad Prism 5.01 (Graph Pad Software, USA). Data were compared using paired (unstimulated versus stimulated) and unpaired (all other comparisons) Student's *t*-tests or a Mann-Whitney* U *test if data were not normally distributed. A *P* value less than 0.05 was considered as statistically significant.

## 3. Results

### 3.1. Baseline Gene Expression in Monocytes

Baseline gene expression was evaluated in peripheral blood monocytes from healthy subjects and APS patients using qRT-PCR. Freshly isolated monocytes from APS patients exhibited downregulated expression of investigated genes. Expression of TLR2 (controls versus APS (mean ± SD); 0.107 ± 0.033 versus 0.026 ± 0.014, *P* = 0.03), IL-1*β* (1.690 ± 0.467 versus 0.394 ± 0.09, *P* = 0.002), IL-6 (0.107 ± 0.046 versus 0.004 ± 0.001, *P* = 0.020), IL-23 (0.125 ± 0.068 versus 0.017 ± 0.005, *P* = 0.030), CCL2 (0.076 ± 0.027 versus 0.008 ± 0.002, *P* = 0.018), and STAT3 (0.164 ± 0.072 versus 0.029 ± 0.018, *P* = 0.028) was significantly higher in monocytes from healthy subjects compared with APS patients. The only exception was CXCL10, with mRNA levels being higher in APS monocytes than in healthy ones (0.1788 ± 0.163 versus 0.96 ± 0.258, *P* = 0.02). No significant differences were observed in mRNA levels for the* NLRP3* and* P2X7* genes in freshly isolated monocytes from both groups (data not shown).

### 3.2. LPS-Induced Expression

**Figure 1:**
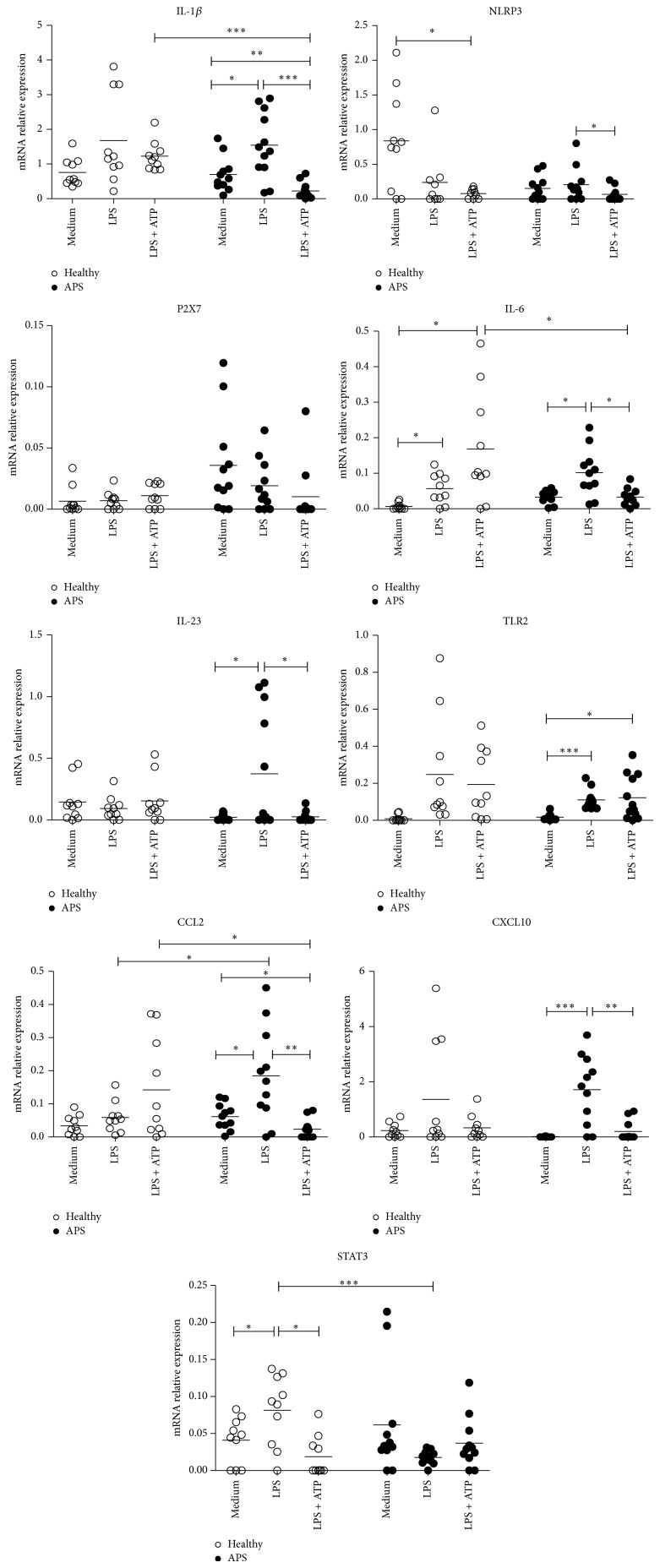
Relative mRNA levels of candidate genes in monocytes from healthy control subjects (healthy) and patients with APS (APS) after 4-hour culture with media alone as a control (media), LPS (10 ng/mL), or LPS + ATP (100 *μ*M) measured by quantitative RT-PCR. All gene expression is relative to RPL32. Data are represented as scatterplots. Horizontal bars represent the mean values for each group (^*^ *P* < 0.05, ^**^ *P* < 0.01, and ^***^ *P* < 0.001).

LPS is the principal component of the outer membrane of Gram-negative bacteria and classic activator for immune cells. To investigate the effects of endotoxin administration on* in vitro* expression of a range of inflammation-related genes, monocytes were incubated with LPS for a period of 4 hours. In monocytes isolated from healthy donors, LPS induced expression of IL-6 (untreated versus LPS-treated; 0.004 ± 0.003 versus 0.055 ± 0.010, *P* = 0.010) and STAT3 (0.040 ± 0.010 versus 0.085 ± 0.017, *P* = 0.040). IL-1*β*, IL-23, TNF-*α*, CXCL10, CCL2, NLRP3, TLR2, and P2X7 gene expressions in monocytes from healthy donors were not induced by LPS (Figure 1).

Upon 4-hour treatment of APS monocytes with LPS, the transcriptional levels of TLR2 (untreated versus LPS-treated; 0.017 ± 0.008 versus 0.113 ± 0.017, *P* = 0.0007), IL-23 (0.016 ± 0.006 versus 0.374 ± 0.138, *P* = 0.02), CCL2 (0.058 ± 0.012 versus 0.183 ± 0.048, *P* = 0.02), CXCL10 (0.009 ± 0.003 versus 1.645 ± 0.409, *P* = 0.0009), IL-1*β* (0.702 ± 0.15 versus 1.543 ± 0.271, *P* = 0.015), and IL-6 (0.028 ± 0.009 versus 0.098 ± 0.021, *P* = 0.03) were elevated as compared with untreated cells. No effect of LPS on NLRP3, STAT3, and P2X7 expression was observed in APS monocytes (Figure 1).

Subsequently, differences in LPS-induced gene expression were determined between both healthy and diseased groups. After LPS stimulation, monocytes from healthy subjects produced higher amounts of STAT3 (healthy versus APS; 0.085 ± 0.017 versus 0.017 ± 0.003, *P* = 0.0007) compared to APS cells, while CCL2 mRNAs (0.05 ± 0.01 versus 0.183 ± 0.048, *P* = 0.03) were higher in monocytes isolated from APS patients. For the remaining genes, significant changes were not detected (*P* > 0.05) (Figure 1).

### 3.3. LPS + ATP-Induced Expression

Several studies showed that LPS-activated monocytes/macrophages require a second stimulus, such as ATP, which acts as an agonist of IL-1 posttranslational processing to produce ultimate amounts of IL-1. Monocytes from both investigated groups were routinely primed with combination of 10 ng/mL LPS and 1 *μ*M ATP for 4 hours. In the healthy group, double stimulation by LPS and ATP increased mRNA levels of IL-6 (untreated versus LPS + ATP-treated; 0.004 ± 0.003 versus 0.163 ± 0.059, *P* = 0.02). The opposite effect of combined stimulation was observed for NLRP3 (0.779 ± 0.317 versus 0.076 ± 0.026, *P* = 0.034). For the remaining genes, significant changes were not detected (Figure 1).

In APS monocytes, double stimulation decreased mRNAs of CCL2 (0.058 ± 0.012 versus 0.023 ± 0.008, *P* = 0.03) and IL-1*β* (0.702 ± 0.150 versus 0.224 ± 0.066, *P* = 0.006) compared to those in control culture with media. By contrast, ATP + LPS increased expression of TLR2 (0.017 ± 0.008 versus 0.122 ± 0.034, *P* = 0.03) whose levels only tended to be significantly increased. For the remaining genes, significant changes were not detected (*P* > 0.05) (Figure 1).

The differences in LPS + ATP-induced mRNAs between monocytes isolated from healthy and APS subjects were analyzed. In cells isolated from healthy donors, the expression of CCL2 (healthy versus APS; 0.136 ± 0.052 versus 0.023 ± 0.008, *P* = 0.03), IL-1*β* (1.239 ± 0.147 versus 0.224 ± 0.066, *P* = 0.0001), and IL-6 (0.163 ± 0.059 versus 0.033 ± 0.008, *P* = 0.03) was higher than in APS monocytes.

### 3.4. ATP Input

The addition of low concentrations of ATP markedly reduced the mRNA levels of a number of genes in APS patients in comparison to those cells cultured only with LPS: IL-1*β* (1.543 ± 0.271 versus 0.224 ± 0.066, *P* = 0.0001), IL-6 (0.098 ± 0.02 versus 0.033 ± 0.008, *P* = 0.017), NLRP3 (0.207 ± 0.073 versus 0.064 ± 0.035, *P* = 0.014), IL-23 (LPS versus LPS + ATP; 0.374 ± 0.138 versus 0.030 ± 0.015, *P* = 0.04), and CCL2 (0.183 ± 0.048 versus 0.023 ± 0.008, *P* = 0.002). The opposite effect was detected for CXCL10 (1.645 ± 0.409 versus 0.204 ± 0.110, *P* = 0.002) whose mRNAs were higher in LPS-induced cells. In monocytes from healthy subjects, STAT3 (0.085 ± 0.017 versus 0.031 ± 0.014, *P* = 0.03) was lower in LPS + ATP-induced cells compared with LPS-induced ones (Figure 1).

### 3.5. Production of IL-1*β* and IL-10 in Supernatants

**Figure 2:**
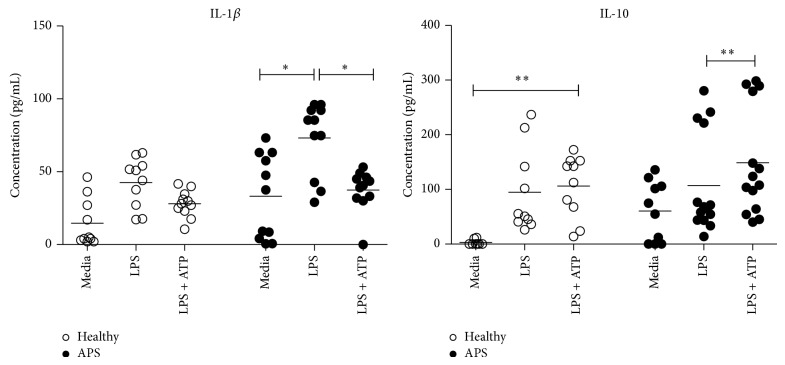
Secreted levels of cytokines IL-1*β* and IL-10 from healthy control subjects (healthy) and patients with APS after 4-hour culture with media alone as a control (media), LPS (10 ng/mL), or LPS + ATP (100 *μ*M) measured by ELISA. Data are represented as scatterplots. Horizontal bars represent the mean values for each group (^*^ *P* < 0.05, ^**^ *P* < 0.01, and ^***^ *P* < 0.001).

To investigate the capacity of monocytes isolated from healthy and diseased subjects to translate pro-IL-1*β* into its mature form IL-1*β*, its content in the culture media was measured using ELISA. In APS monocytes, LPS increased production of IL-1*β* (33.88 ± 18.49 versus 65.26 ± 10.70 pg/mL, *P* = 0.048) compared with control culture. By contrast, double stimulation decreased production of IL-1*β* (65.26 ± 10.07 versus 38.08 ± 5.213 pg/mL, *P* = 0.041) in APS monocytes when compared to LPS stimulation. In the healthy group, production of IL-1*β* by monocytes was not affected by administration of LPS and LPS + ATP (Figure 2).

To test whether ATP exerted its anti-inflammatory effect on LPS-activated cells, we measured IL-10 protein in supernatants. Monocytes secreted high amounts of IL-10 after exposure of the cells with LPS + ATP in both investigated groups. In APS monocytes double stimulation increased production of IL-10 (143.7 ± 38.76 pg/mL versus 111.2 ± 39.62, *P* = 0.008) compared with LPS cultivation; in monocytes from healthy donors double stimulation resulted in increased production of IL-10 (114.4 ± 27.31 versus 2.988 ± 2.988, *P* = 0.008) compared with control cultivation (Figure 2).

## 4. Discussion

Autoimmune diseases have a multifactorial etiology depending on both genetic and environmental factors [27]. Association of thrombotic events with various infections in patients with APS has also been recognized [28]. Currently, mechanisms by which microbial agents trigger autoimmune reaction include induction of pathogenic autoantibody production as a result of molecular mimicry and cross reactivity [29] and polyclonal activation of distinct T cell subclasses [27]. Monocytes are crucial players in sensing pathogens as well as priming and maintenance of adaptive responses [14, 30]. Particularly, monocytes were shown to play an essential role in the development of T cell response [31] and contribute to the initiation and maintenance of autoresponse inflammation. It was therefore of significant interest to explore the features of monocyte activation by bacterial ligands in APS.

Our experiments showed significant differences in expression of several genes in monocytes from APS patients that may be important to proinflammatory potential of the cells compared with gene expression in monocytes isolated from healthy controls. Incubation of APS cells with LPS resulted in increased expression of the following genes: IL-1*β*, IL-6, IL-23, TLR2, CCL2, and CXCL10, while the cells from healthy donors were less responsive to LPS (Figure 1). The findings indicate increased sensitivity of APS cells to LPS that may enhance development or progression of autoimmune processes in APS.

Accumulation of activated monocytes in the inflamed sites is widely recognized to play an inflammatory and tissue destructive role [32]. Furthermore, transmigration of activated monocytes through the venous and arterial walls is a key event in thrombus formation, the most common complications associated with APS [10]. An important mechanism of hypercoagulability in APS is increased expression of TF by monocytes [11]. In addition, there are substantial* in vitro* and* in vivo* data supporting the important role of monocyte-derived proinflammatory cytokines in mediating thrombogenesis. Increased production of proinflammatory cytokines, chemokines, and adhesion molecules, such as IL-6, TNF-*α*, MCP-1, TGF-*β*, and IL-8, may significantly increase the risk for thrombotic events [33]. The importance of monocyte-mediated inflammation in induction of the procoagulation cascade was observed in various prothrombotic conditions [34, 35]. However, molecular mechanisms of thrombus formation as well as the mechanisms linking inflammation and thrombosis in APS are still elusive. Thus, enhanced ability of APS monocytes to alter their phenotype in the presence of LPS may substantially contribute to thrombus formation in APS patients.

Monocyte-derived cytokines are also able to attract and activate different subsets of lymphocytes, such as Th17 cells, which mediate host defense against extracellular bacteria and fungi. Th17 differentiation or expansion requires the presence of IL-1*β*, IL-6, TGF-*β*, and IL-23 [36, 37]. Inappropriate Th17 activation was shown to play a crucial role in the induction/maintenance of multiple types of diseases, including autoimmune tissue injury [38]. Thus, the shifted phenotype of LPS-induced APS monocytes observed in our study may contribute to the autoimmune responses in APS.

While LPS promotes synthesis of large quantities of pro-IL-1*β*, efficient posttranslational processing requires a secondary stimulus such as ATP [39]. ATP is classic intracellular metabolite playing crucial roles in energy metabolism and electron transfer. In recent years, it has been recognized that extracellular ATP, released from intracellular stores in response to cellular stress or inflammation, may function as a “danger” signal which modulates response of the innate immune system [40, 41]. Once outside of the cell, ATP functions as a potent regulatory molecule and modulates a broad range of cell functions in a dose-dependent manner [42, 43]. While several studies describe ATP as a signal that triggers and maintains inflammation, others suggest that ATP represents a negative feedback signal to limit detrimental inflammation in affected cells. According to our results, stimulation with ATP failed to trigger further increase in expression of LPS-stimulated IL-1*β*. Moreover, the presence of ATP even suppressed LPS-induced IL-1*β* expression in APS monocytes. Our results show that reduction in IL-1*β* expression occurs via the NLRP3 pathway as a result of reduced NLRP3 protein expression. In normal cells, the same tendency was observed (Figure 1). Similarly to what was observed for IL-1*β* and NLRP3, expression of IL-6, IL-23, CCL2, and CXCL10 was inhibited by ATP in a significant way in monocytes from APS patients but not in the healthy subjects. It is noteworthy to mention that ATP reduces LPS-induced mRNA levels of above-mentioned mediators until the baseline levels, which are significantly lower in diseased group compared with those in healthy one (data not shown). We also confirmed upregulated expression of IL-1*β* in LPS-induced and downregulated expression of IL-1*β* in LPS + ATP-induced APS cells by measuring IL-1*β* protein production (Figure 2). Thus, our findings are consistent with the recently defined anti-inflammatory effect of ATP at low concentrations [44], which in case of APS is more profound.

ATP mediates its diverse effects by binding to and activating a broad range of cell surface P2Y and P2X receptors [45]. In particular, the P2X7 receptor has attracted considerable interest since its activation triggers maturation and release of proinflammatory IL-1*β* in monocytes and macrophages [46]. Our results did not reveal any obvious differences in P2X7 mRNA levels in both investigated groups in the presence of LPS or LPS + ATP. Thus, low ATP concentrations used in the present study were not sufficient to activate the P2X7 receptor and exert its proinflammatory properties. These findings strengthen the anti-inflammatory mechanism of low ATP concentrations in APS and healthy monocytes. Anti-inflammatory properties of ATP could be mediated not only via P2X7 receptor but also via other purinergic receptors. La Sala and colleagues have shown that chronic exposure to low-dose ATP stimulated macrophages to secrete anti-inflammatory cytokines suppressing inflammation and contributed to Th2 development. Particularly, activation of P2Y12 or P2Y11 by ATP suppresses LPS-induced production of proinflammatory cytokines such as IL-1*β*, IL-6, and TNF-*α* and potentiates IL-10 [47].

Whether extracellular ATP is able to trigger production of immunosuppressive factors is still a debated issue. We tried to confirm this observation by measuring IL-10 in culture supernatants (Figure 2). IL-10 is a potent Th2-driving anti-inflammatory cytokine with diverse immunomodulatory activity, including the inhibition of proinflammatory cytokine release and antigen presentation [48, 49]. Very recently, it was shown that IL-10 may regulate IL-1*β* secretion and inflammasome activity by suppressing gene expression of inflammasome components [50]. Therefore, we suggest that IL-10, which is produced to counterregulate the inflammatory response, would also be able to provide a degree of immunomodulation capable of inhibiting unnecessary tissue injury and progression of autoimmune reactions.

In conclusion, we provided evidence of enhanced ability of monocytes from APS patients to alter their phenotype in the presence of low concentrations of endogenous and exogenous ligands. APS monocytes are overactivated in the presence of LPS, and this overactivation is suppressed by ATP, which supports the hypothesis that extracellular ATP is a crucial immunomodulating agent. Our results suggest that ATP blocks as yet unknown signal induced by LPS that contributes to anti-inflammatory ATP-mediated suppression of inflammasome-dependent IL-1*β* expression and elevated IL-10 production. We can speculate that such mechanisms would enable populations of circulating monocytes to effectively control proinflammatory signals and evoke immunoregulatory mechanisms that protect from tissue injury and progression of autoimmune reactions. Current knowledge on mechanisms involved in the induction of autoimmune mechanisms of APS as well as contribution of monocytes to APS pathogenesis is emerging. Therefore, continued efforts in this direction are required. Understanding of the innate mechanisms responsible for environmentally induced autoimmunity will produce new information on the processes that drive autoimmunity.

## Supplementary Material

Table S1: Description of investigated genes and used primers.
