# Supplementary material for: Differential Regulation of Proinflammatory Mediators following LPS- and ATP-Induced Activation of Monocytes from Patients with Antiphospholipid Syndrome
Source: Biomed Res Int. 2015 Feb 15;2015:292851. doi: 10.1155/2015/292851 (PMC4345072; doi:10.1155/2015/292851)
Supplement: Supplementary file 1 — Table S1: Description of investigated genes and used primers. [file 292851.f1.pdf]

Table S1: Description of investigated genes and used primers.

Gene abbreviation	Gene name (synonyms)	GenBank[a] Accession number	Sense, antisense primers	LNA probe[b]
RPL32[c]	Ribosomal protein L32	NM_000994.3	5´- gaa gtt cct ggt cca caa cg -3´L<br>5´- gcg atc tcg gca cag taa g -3´ R	#17
IL-1B[c]	Interleukin 1, beta	NM_000576.2	5´- tac ctg tcc tgc gtg ttg aa-3´ L<br>5´- tct ttg ggt aat ttt tgg gat ct-3´ R	#78
NLRP3[c]	NLR family, pyrin domain containing 3	NM_001243133.1	5´- tgt cct ccc aag ctc ctc t -3´L<br>5´- aag cag cac tca tgc gag a -3´ R	#27
CCL2[c]	Chemokine (C-C motif) ligand 2	NM_002982.3	5´- agt ctc tgc cgc cct tct -3´L<br>5´- gtg act ggg gca ttg att g-3´ R	#40
TLR2[d]	Toll-like receptor 2	NM_003264.3	MW:6364 3202<br>MW:6364 5433	#14
IL-6[d]	Interleukin 6	NM_000600.3	80117B3-0314C03 7/85<br>80117B3-0314D03 8/58	#7
CXCL10[d]	Chemokine (C-X-C motif) ligand 10	NM_001565.3	70221B3-07107F07 30/67<br>70221B3-0396B03 14/57	#34
STAT3[d]	Signal transducer and activator of transcription 3	NM_003150.3	81106B3-0729A07 11/99<br>81106B3-0729B07 9/90	#18
IL-23A[d]	Interleukin 23, alpha subunit p19	NM_016584.2	80117B3-0213G02 3/58<br>80117B3-0213H02 4/58	#76
P2X7[e]	Purinergic receptor P2X, ligand-gated ion channel, 7 (P2RX7)	NM_002562.5	Hs00175721_m1	

[a]Gene sequences available online at http://www.ncbi.nlm.nih.gov/

[b] Numbers of Locked Nucleic Acid (LNA) probes according to the commercially available library (www.universalprobelibrary.com)

[c]Primers/probe sets (PrimeTime qPCR Assays**)** were from Metabion International AG, Germany

[d]Primers/probe sets (PrimeTime qPCR Assays**)** were from Integrated DNA Technologies, Coralville, Iowa, USA

[e]Primers/probe sets (TaqMan® Gene Expression Assay) were from Life Technologies Corporation, Applied Biosystem Carlsbad, USA
